# CO_2_ Reduction Using Water as an Electron Donor over Heterogeneous Photocatalysts Aiming at Artificial Photosynthesis

**DOI:** 10.1021/acs.accounts.1c00676

**Published:** 2022-03-01

**Authors:** Shunya Yoshino, Tomoaki Takayama, Yuichi Yamaguchi, Akihide Iwase, Akihiko Kudo

**Affiliations:** Department of Applied Chemistry, Faculty of Science, Tokyo University of Science, 1-3 Kagurazaka, Shinjuku-ku, Tokyo 162-8601, Japan

## Abstract

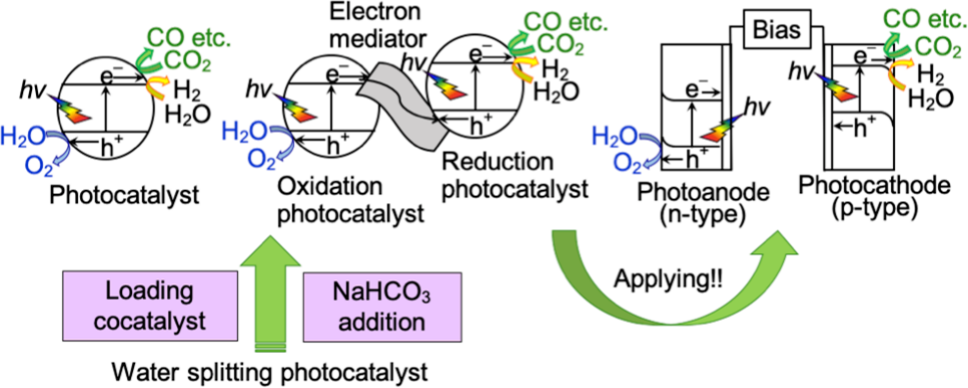

Photocatalytic and photoelectrochemical CO_2_ reduction of artificial photosynthesis is a promising chemical process to solve resource, energy, and environmental problems. An advantage of artificial photosynthesis is that solar energy is converted to chemical products using abundant water as electron and proton sources. It can be operated under ambient temperature and pressure. Especially, photocatalytic CO_2_ reduction employing a powdered material would be a low-cost and scalable system for practical use because of simplicity of the total system and simple mass-production of a photocatalyst material.

In this Account, single particulate photocatalysts, Z-scheme photocatalysts, and photoelectrodes are introduced for artificial photosynthetic CO_2_ reduction. It is indispensable to use water as an electron donor (i.e., reasonable O_2_ evolution) but not to use a sacrificial reagent of a strong electron donor, for achievement of the artificial photosynthetic CO_2_ reduction accompanied by Δ*G* > 0. Confirmations of O_2_ evolution, a ratio of reacted e^–^ to h^+^ estimated from obtained products, a turnover number, and a carbon source of a CO_2_ reduction product are discussed as the key points for evaluation of photocatalytic and photoelectrochemical CO_2_ reduction.

Various metal oxide photocatalysts with wide band gaps have been developed for water splitting under UV light irradiation. However, these bare metal oxide photocatalysts without a cocatalyst do not show high photocatalytic CO_2_ reduction activity in an aqueous solution. The issue comes from lack of a reaction site for CO_2_ reduction and competitive reaction between water and CO_2_ reduction. This raises a key issue to find a cocatalyst and optimize reaction conditions defining this research field. Loading a Ag cocatalyst as a CO_2_ reduction site and NaHCO_3_ addition for a smooth supply of hydrated CO_2_ molecules as reactant are beneficial for efficient photocatalytic CO_2_ reduction. Ag/BaLa_4_Ti_4_O_15_ and Ag/NaTaO_3_:Ba reduce CO_2_ to CO as a main reduction reaction using water as an electron donor even in just water and an aqueous NaHCO_3_ solution. A Rh–Ru cocatalyst on NaTaO_3_:Sr gives CH_4_ with 10% selectivity (Faradaic efficiency) based on the number of reacted electrons in the photocatalytic CO_2_ reduction accompanied by O_2_ evolution by water oxidation.

Visible-light-responsive photocatalyst systems are indispensable for efficient sunlight utilization. Z-scheme systems using CuGaS_2_, (CuGa)_1–*x*_Zn_2*x*_S_2_, CuGa_1–*x*_In_*x*_S_2_, and SrTiO_3_:Rh as CO_2_-reducing photocatalyst, BiVO_4_ as O_2_-evolving photocatalyst, and reduced graphene oxide (RGO) and Co-complex as electron mediator or without an electron mediator are active for CO_2_ reduction using water as an electron donor under visible light irradiation. These metal sulfide photocatalysts have the potential to take part in Z-scheme systems for artificial photosynthetic CO_2_ reduction, even though their ability to extract electrons from water is insufficient.

A photoelectrochemical system using a photocathode is also attractive for CO_2_ reduction under visible light irradiation. For example, p-type CuGaS_2_, (CuGa)_1–*x*_Zn_2*x*_S_2_, Cu_1–*x*_Ag_*x*_GaS_2_, and SrTiO_3_:Rh function as photocathodes for CO_2_ reduction under visible light irradiation. Moreover, introducing a conducting polymer as a hole transporter and surface modification with Ag and ZnS improve photoelectrochemical performance.

## Key References

- IizukaK.; WatoT.; MisekiY.; SaitoK.; KudoA.Photocatalytic Reduction of Carbon Dioxide over Ag Cocatalyst-Loaded ALa_4_Ti_4_O_15_ (A = Ca, Sr, and Ba) Using Water as a Reducing Reagent. J. Am. Chem. Soc.2011, 133, 20863–208682208785610.1021/ja207586e.^1^ Ag-cocatalyst for an effective active site for CO_2_ reduction and Ag/BaLa_4_Ti_4_O_15_ photocatalyst for CO_2_ reduction to form CO as a main reduction product using water as an electron donor even in an aqueous solution.
- NakanishiH.; IizukaK.; TakayamaT.; IwaseA.; KudoA.Highly Active NaTaO_3_-Based Photocatalysts for CO_2_ Reduction to Form CO Using Water as the Electron Donor. ChemSusChem2017, 10, 112–1182787426910.1002/cssc.201601360.^2^ Ag/NaTaO_3_ photocatalyst doped with alkaline earth cations for CO_2_ reduction to CO with 90% of selectivity in an aqueous solution with a basic salt for enhancement of hydrated CO_2_ molecules supply.
- IwaseA.; YoshinoS.; TakayamaT.; NgY. H.; AmalR.; KudoA.Water Splitting and CO_2_ Reduction under Visible Light Irradiation Using Z-Scheme Systems Consisting of Metal Sulfides, CoOx-Loaded BiVO_4_, and a Reduced Graphene Oxide Electron Mediator. J. Am. Chem. Soc.2016, 138, 10260–102642745902110.1021/jacs.6b05304.^3^ Z-scheme system composed of CuGaS_2_ as a reducing photocatalyst and RGO–(CoO_*x*_/BiVO_4_) as an O_2_-evolving photocatalyst for CO_2_ reduction to CO using water as an electron donor under visible light irradiation in an aqueous powder suspension system.
- YoshinoS.; IwaseA.; YamaguchiY.; SuzukiT. M.; MorikawaT.; KudoA.Photocatalytic CO_2_ Reduction Using Water as an Electron Donor under Visible Light Irradiation by Z-Scheme and Photoelectrochemical Systems over (CuGa)_0.5_ZnS_2_ in the Presence of Basic Additives. J. Am. Chem. Soc.2022, 144, 2323–23323507623010.1021/jacs.1c12636PMC8832390.^4^ Employing (CuGa)_0.5_ZnS_2_ prepared by a flux method in Z-scheme and photoelectrochemical systems with tuning a reactant solution for efficient and stable CO_2_ reduction to form CO with 10–20% selectivity using water as an electron donor under visible light.

## 1 Introduction

Carbon dioxide capture storage and utilization technology (CCSU) has been encouraged, because CO_2_ emission control is a critical issue in the world. Ideally, CO_2_ fixation should be realized utilizing renewable energies, such as solar energy, as follows:

- hydrogenation of CO_2_ using solar hydrogen
- biological CO_2_ fixation
- electrochemical CO_2_ reduction utilizing a photovoltaic cell
- photocatalytic and photoelectrochemical CO_2_ reduction directly utilizing solar light

There are advantages and disadvantages to each reaction. Hydrogenation of CO_2_ can produce various beneficial chemical compounds with high CO_2_ conversion efficiency through a thermal catalytic process on an industrial scale. Much knowledge toward CO_2_ hydrogenation has been accumulated in C1 chemistry so far. The hydrogen should be supplied from solar hydrogen production by water splitting with no consumption of fossil resources and no CO_2_ emission but not from steam reforming of fossil resources. However, the CO_2_ conversion process requires high temperature and pressure to operate the catalytic process. Biological CO_2_ fixation is based on natural photosynthesis by plants. Natural photosynthesis involves almost no energy loss for absorbed photon energy conversion. However, the solar energy conversion efficiency is limited because a plant absorbs only a part of the solar spectrum as indicated by its green color. Electrochemical CO_2_ reduction is also interesting from the viewpoint of electrocatalysis. The reduction products and the selectivity change with electrode materials even under the same electrolysis conditions. However, electrolyzers and batteries are indispensable for the electrochemical system in addition to a photovoltaic cell. Photocatalytic and photoelectrochemical CO_2_ reduction utilizing solar energy in an aqueous solution is one of the ideal chemical reactions for artificial photosynthesis, because solar energy is directly converted and stored as chemical products. Artificial photosynthesis can be operated under ambient temperature and pressure to produce solar fuels and chemicals and can exceed natural photosynthesis in solar energy conversion efficiency. Especially, a powder-based photocatalyst is attractive because it can be employed for a low-cost and scalable system aimed at artificial photosynthesis.^5^

In this Account, we introduce several types of CO_2_ reduction systems, mainly based on particulate photocatalyst materials, using water as an electron donor. Key points for evaluation of photocatalytic and photoelectrochemical CO_2_ reduction are also discussed.

## 2 Overview of Photocatalytic and Photoelectrochemical CO_2_ Reduction Systems for Artificial Photosynthesis

**Figure 1:**
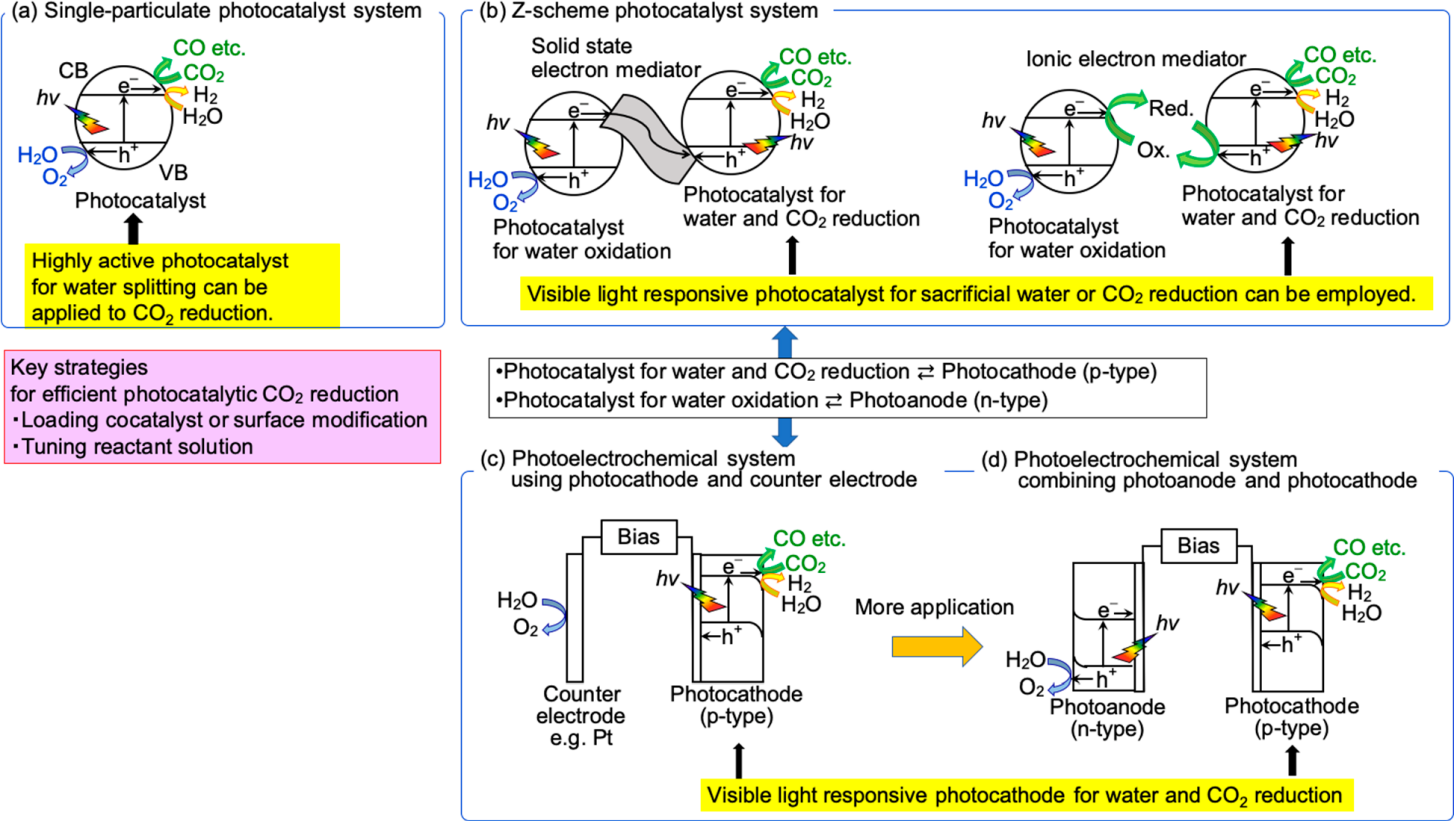
Artificial photosynthetic CO_2_ reduction based on a powdered photocatalyst by (a) a single-particulate system, (b) a Z-scheme system, (c) a photoelectrochemical system using a photocathode, and (d) a photoelectrochemical system combining a photocathode and a photoanode.

Figure 1 shows various types of photocatalytic and photoelectrochemical systems for artificial photosynthesis.^6,7^ The first is a single-particulate photocatalyst system via one-photon excitation, in which photocatalytic reduction by photogenerated electrons and photocatalytic oxidation by photogenerated holes proceed on one particle (Figure 1a).^6−9^ Photocatalysts of semiconductor materials have a band structure in which a conduction band (CB) is separated from a valence band (VB) with a band gap (BG). The thermodynamic relationship between the band structure of a photocatalyst and the redox potential for the objective reaction is important. The equilibrium potentials relative to the normal hydrogen electrode (NHE) at pH 7 and 298 K for CO_2_ reduction and water splitting are as follows:
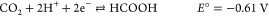
1
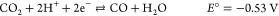
2
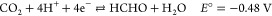
3
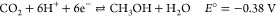
4
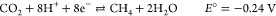
5
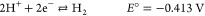
6
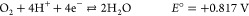
7The conduction band minimum and valence band maximum should locate at more negative and positive levels than redox potentials of objective reactions such as water splitting and CO_2_ reduction, respectively. When the energy of the incident photon is larger than that of the band gap, electrons and holes are photogenerated in the conduction band and the valence band, respectively. The photogenerated electrons reduce water and CO_2_ to generate H_2_ and CO_2_ reduction products such as CO, while the photogenerated holes oxidize water to form O_2_. The O_2_ evolution is a key issue for photocatalytic CO_2_ reduction using water as an electron donor. Moreover, since CO_2_ reduction competes with water reduction, selective CO_2_ reduction is also challenging from the viewpoints of not only thermodynamics but also kinetics. Therefore, the catalytic ability of photocatalyst surface is also a key issue.

The second is a Z-scheme system via a two-photon excitation process consisting of a reducing photocatalyst, an oxidizing photocatalyst, and an electron mediator (Figure 1b).^10−13^ This system mimics natural photosynthesis by a plant. Various photocatalysts that are active for either photocatalytic reduction of water and CO_2_ reduction or photocatalytic oxidation of water can be employed to make a Z-scheme system. From this viewpoint, it is meaningful to test photocatalytic CO_2_ reduction using sacrificial electron donors such as organic compounds and S^2–^ in order to find potential CO_2_-reducing photocatalysts in a part of the Z scheme system, though the sacrificial reaction becomes a downhill reaction (Δ*G* < 0).

The third is a photoelectrochemical cell.^7^ n-Type and p-type semiconductors may function as O_2_-evolving photoanodes and photocathodes to give H_2_ and reduction products of CO_2_, respectively. The photoelectrochemical cell can be constructed by combining a photoelectrode of a working electrode with a counter electrode (Figure 1c) or combining a photoanode and a photocathode working via two-photon excitation (Figure 1d). External bias can be applied between the photoanode and photocathode to enhance the photoelectrochemical reaction. However, the external bias should be smaller than the theoretical voltage of electrolysis of an objective reaction to achieve artificial photosynthesis from light energy conversion.

In the following sections, several types of the photocatalytic and photoelectrochemical systems shown in Figure 1 are introduced.

## 3 Single Particulate Photocatalysts with Wide Band Gaps for CO_2_ Reduction Using Water as an Electron Donor (Figure 1a)

### 3.1 Ag Cocatalyst for CO Formation by Photocatalytic CO_2_ Reduction

CO_2_ reduction over metal oxide photocatalysts has extensively been investigated. Although TiO_2_ has widely been studied for photocatalytic CO_2_ reduction, those reports involve critical issues such as lack of quantification of O_2_ and small amounts of reduction products such as CH_4_ due to low activities. Ishitani et al. reported that CH_4_ could come from contaminants adsorbed on TiO_2_.^14^ In contrast, Sayama and Arakawa have reported that a ZrO_2_ photocatalyst (BG = 5.0 eV) produced CO, H_2_, and O_2_ in stoichiometric amounts in an aqueous medium.^15^ Moreover, loading a Cu cocatalyst and adding a bicarbonate ion enhanced the photocatalytic CO_2_ reduction. This is the first report to demonstrate photocatalytic CO_2_ reduction using water as an electron donor over a particulate photocatalyst. However, the major reduction product was H_2_ and the selectivity for CO formation (CO/(H_2_ + CO)) was about 12%. In such a background, the author found a highly active Ag cocatalyst for photocatalytic CO_2_ reduction to form CO with highly active photocatalysts for water splitting.

**Figure 2:**
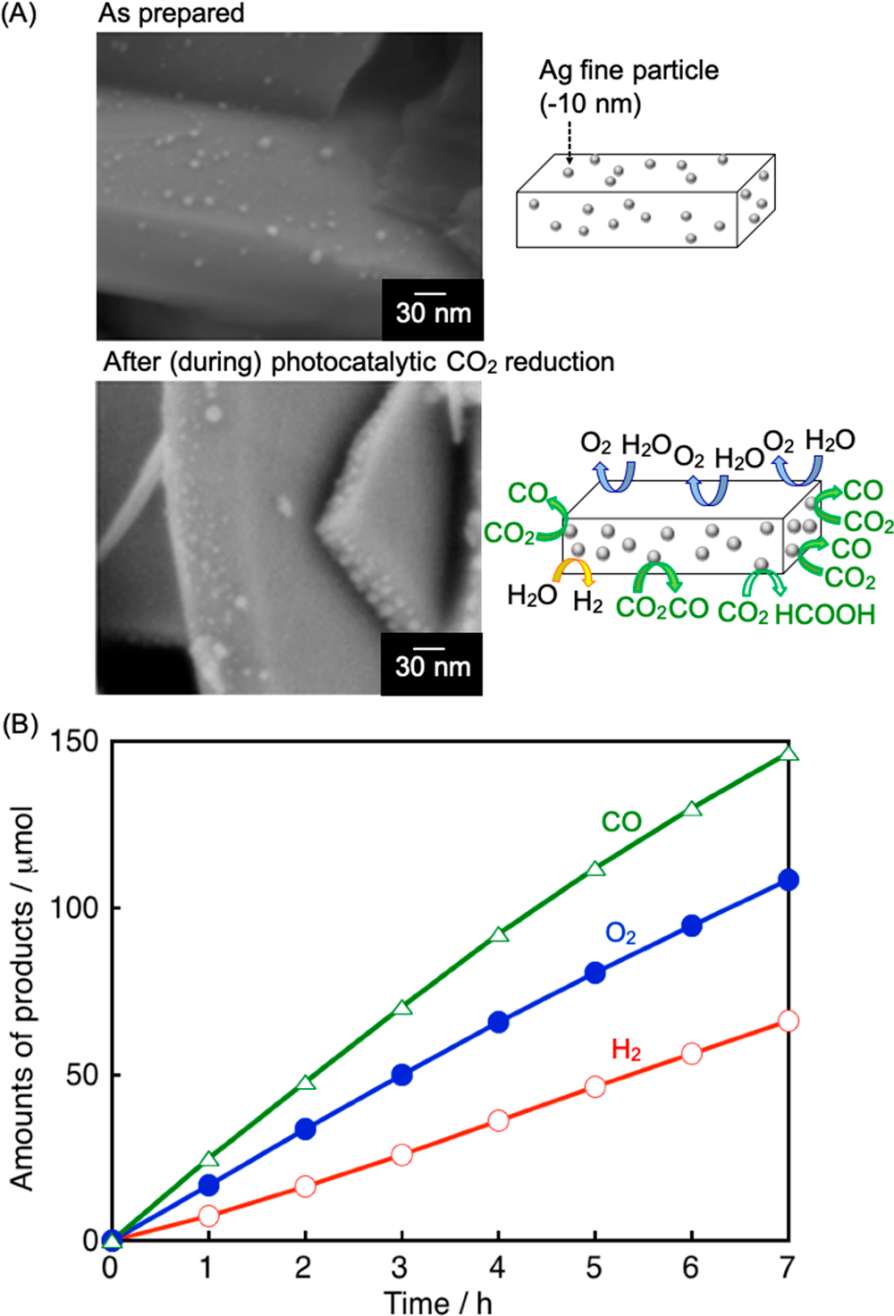
(A) SEM images of Ag/BaLa_4_Ti_4_O_15_ before and after photocatalytic CO_2_ reduction, and the proposed mechanism. (B) Photocatalytic CO_2_ reduction using water as an electron donor under UV light irradiation over Ag(2 wt %)/BaLa_4_Ti_4_O_15_.^1^ Photocatalyst, 0.3 g; reactant solution, water (360 mL); flow gas, CO_2_ (1 atm); light source, 400 W high-pressure mercury lamp; reaction cell, inner irradiation quartz cell. Reproduced with permission from ref (1). Copyright 2011 American Chemical Society.

BaLa_4_Ti_4_O_15_ (BG = 3.9 eV) photocatalyst with a layered perovskite structure was first chosen because NiO_*x*_/BaLa_4_Ti_4_O_15_ efficiently split water.^16^ The particle is plate shaped in which an edge plane and a basal plane are reduction and oxidation site, respectively, as shown in Figure 2A. The separation of the reduction site from the oxidation site is beneficial for an uphill reaction, because a back reaction of a downhill reaction is suppressed. Ag was found to be a highly active cocatalyst for photocatalytic CO_2_ reduction to form CO as shown in Table 1.^1^ To compare photocatalytic CO_2_ reduction abilities, not only the production rate [mol h^–1^] but also the selectivity for CO_2_ reduction are essential values. The selectivity is calculated according to eq 8.
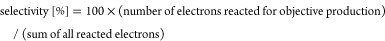
8The selectivity and the production rate based on the number of reacted electrons are similar to Faradaic efficiency and partial current density, respectively, in an electrochemical reaction. It is noteworthy that CO is the main reduction product with about 70% selectivity, rather than H_2_, even in an aqueous medium (Table 1). A small amount of HCOOH was also obtained. It is reasonable that Ag functions as an efficient cocatalyst to form CO judging from its electrocatalysis in aqueous CO_2_ solution.^17,18^ The high conduction band level of BaLa_4_Ti_4_O_15_ should be important to get an enough driving force for CO_2_ reduction and high energy potential of photogenerated electrons applied to the Ag cocatalyst. A liquid-phase reduction method gives higher activity for CO formation than photodeposition and impregnation methods for the Ag cocatalyst loading. There is concern that the Ag cocatalyst may efficiently reduce O_2_ produced by water splitting. However, O_2_ reduction on the Ag cocatalyst is suppressed more or less, because the reaction is conducted under CO_2_ flow conditions smoothly removing the O_2_ from the reaction system.

**Table 1:** Effect of Cocatalyst on CO_2_ Reduction Using Water as an Electron Donor under UV Light Irradiation over BaLa_4_Ti_4_O_15_ Photocatalyst^1^^a^

		activity [μmol h^–1^]					
cocatalyst (wt %)	loading method	H_2_	O_2_	CO	HCOOH	CO selectivity (%)	e^–^/h^+^
none		5.3	2.4	0	0	0	1.1
NiO_*x*_ (0.5)	impregnation^b^	58	29	0.02	0	0.03	1.0
Ru (0.5)	photodeposition	84	41	0	0	0	1.0
Cu (0.5)	photodeposition	96	45	0.6	0	0.6	1.1
Au (0.5)	photodeposition	110	51	0	0	0	1.1
Ag (1.0)	photodeposition	10	7.0	4.3	0.3	30	1.0
Ag (1.0)	impregnation	8.2	5.7	5.2	0.2	38	1.2
Ag (1.0)	impregnation + H_2_ reduction	5.6	8.7	8.9	0.3	60	0.9
Ag (1.0)	liquid-phase reduction	5.6	12	19	0.4	76	1.0

a Photocatalyst, 0.3 g; reactant solution, water (360 mL); flow gas, CO_2_ (1 atm); light source, 400 W high-pressure mercury lamp; reaction cell, inner irradiation quartz cell.

b Treated with H_2_ reduction and subsequent oxidation.

Figure 2A shows SEM images of Ag-cocatalyst before and after photocatalytic CO_2_ reduction and a reaction mechanism. BaLa_4_Ti_4_O_15_ is a plate-like particle with layered perovskite structure. Ag particles of ∼10 nm diameter are loaded on both edge and basal plane by the liquid-phase reduction as prepared. After photocatalytic CO_2_ reduction, the number of the Ag cocatalyst particles on the edge increases while Ag particles on the basal plane disappear, because Ag on the basal plane dissolves by photooxidation and is subsequently photodeposited on the edge by photoreduction during the photocatalytic CO_2_ reduction.

Figure 2B shows time courses of CO, H_2_, and O_2_ evolution by photocatalytic CO_2_ reduction over Ag/BaLa_4_Ti_4_O_15_. The time courses demonstrate not only activity and durability but also other important points to evaluate photocatalytic CO_2_ reduction as discussed below.

It is important to see if O_2_ evolves in a stoichiometric amount when the photocatalytic reaction is conducted using water as an electron donor for light energy conversion without any strong sacrificial electron donors. CO_2_ is reduced by photogenerated electrons on a photocatalyst, while photocatalytic oxidation of water by photogenerated holes simultaneously proceeds as the counterpart as shown in Figures 1a and 2B. It is also important to see if the ratio of reacted electrons to holes estimated from products is unity according to eq 9.
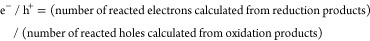
9Unity means that reduction and oxidation products are obtained in a stoichiometric amount. If the e^–^/h^+^ is not unity, side reactions or noncatalytic but quantitative reactions such as reduction or oxidation of the photocatalyst itself may proceed. In addition, it is necessary to pay attention to whether some products are not detected by the measurement technique employed. The O_2_ evolution with at unity e^–^/h^+^ ratio is satisfied for the present photocatalytic CO_2_ reduction over Ag/BaLa_4_Ti_4_O_15_ as shown in Table 1 and Figure 2B.

Photocatalytic reaction must proceed by irradiation the energy of which is larger than the band gap energy. The band gap of BaLa_4_Ti_4_O_15_ is 3.9 eV, which corresponds to about 320 nm light. This photocatalyst works with use of a quartz reaction cell with a suitable UV lamp, while the activity is negligible using a Pyrex reaction cell. This result indicates that the photoresponse of the BaLa_4_Ti_4_O_15_ photocatalyst is reasonable.

Turnover number defined by eq 10 is also an important indicator to consider if the reaction proceeds photocatalytically.
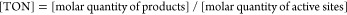
10Turnover number (TON) indicates how many atoms or molecules react on one active site. TON based on the number of reacted electrons is often used for photocatalysis accompanied by redox reactions according to eq 11.
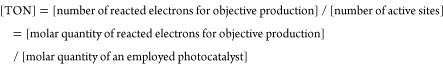
11If the TON is too small, we cannot guarantee that it is a photocatalytic reaction because not catalytic but quantitative reactions on the surface of photocatalyst cannot be excluded. In a heterogeneous photocatalyst, the molar quantity of the active site is often replaced with the molar quantity of an employed photocatalyst, because it is difficult to estimate the number of actual active sites on the surface of a photocatalyst. In some cases, the molar quantities of atoms on the surface, dopant, and cocatalyst are used for the denominator. The TON should be above unity to prove that the reaction proceeds catalytically. Photocatalytic CO_2_ reduction over Ag/BaLa_4_Ti_4_O_15_ proceeds steadily under UV light irradiation and TON to photocatalyst and cocatalyst reach 1.6 and 7.7, respectively, at 7 h being above unity as shown in Figure 2B.

Products of CO and HCOOH among others must originate from CO_2_. However, contaminants on the photocatalyst and some carbon materials constituting the photocatalyst system may become a carbon source.^14,19^ Therefore, confirmation of the carbon source of the obtained products is necessary. One approach is an isotope experiment using ^13^CO_2_. Another is a control experiment using an inert gas to confirm that carbon products are not obtained. The reactant solution conditions (i.e., pH) of the control experiment should be similar to those of CO_2_ reduction. When ^13^CO_2_ is flowed for a photocatalytic reaction over Ag/BaLa_4_Ti_4_O_15_, ^13^CO is obtained while ^12^CO is not. In addition, CO is not obtained when Ar instead of CO_2_ is supplied. These two results prove CO_2_ is the carbon source.

Thus, it is concluded by confirmations of O_2_ evolution, the ratio of reacted e^–^ to h^+^ estimated from obtained products, the TON, and the carbon source that the CO_2_ reduction photocatalytically proceeds using water as an electron donor over Ag/BaLa_4_Ti_4_O_15_.

### 3.2 Effect of HCO_3_^–^ in Water on Photocatalytic CO_2_ Reduction

**Figure 3:**
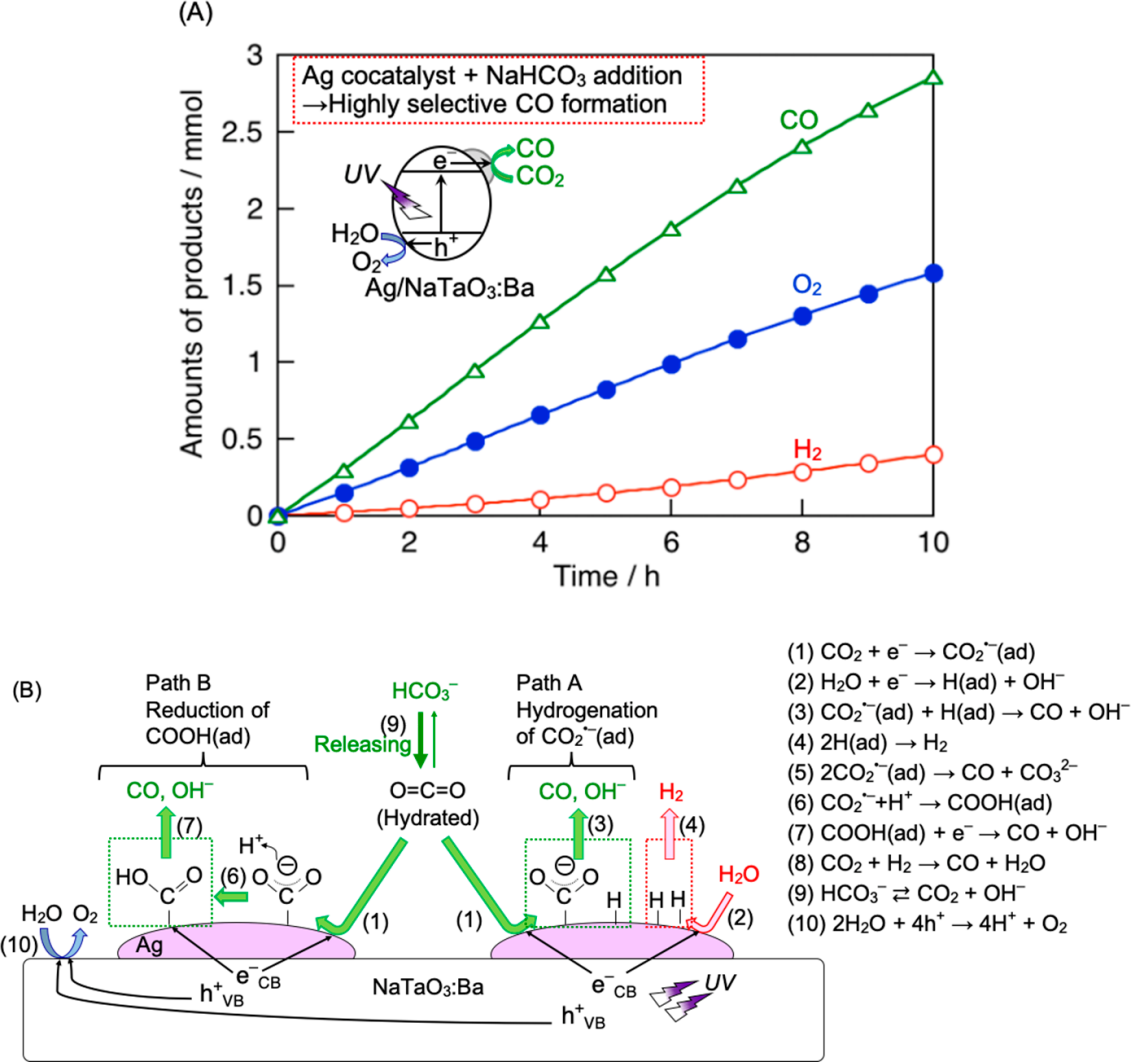
(A) Photocatalytic CO_2_ reduction using water as an electron donor under UV light irradiation over Ag/NaTaO_3_:Ba. Reactant solution, NaHCO_3(aq)_ (360 mL); flow gas, CO_2_ (1 atm); light source, 400 W high-pressure mercury lamp; reaction cell, an inner irradiation quartz cell. (B) Proposed mechanism of photocatalytic CO_2_ reduction in the presence of NaHCO_3_.^2^ Reproduced with permission from ref (2). Copyright 2017 Wiley.

La or alkaline earth metal doped NaTaO_3_ (BG = 4.1 eV) with a perovskite structure is also a unique photocatalyst. The doped NaTaO_3_ has a surface nanostep structure in which a reduction site is separated from an oxidation site.^20,21^ While NiO/NaTaO_3_ with dopant splits water efficiently but does not reduce CO_2_, Ag/NaTaO_3_:Ba gives CO with about 50% selectivity under UV light upon flowing CO_2_ into pure water.^2^ Moreover, with addition of a basic salt into the reactant solution, CO formation rate drastically increases and the selectivity reaches about 90% even in an aqueous solution (Figure 3A). The enhancement of CO_2_ reduction with salt addition is due to efficient supply of hydrated CO_2_ molecule reactant and pH control.

A proposed mechanism of photocatalytic CO_2_ reduction over Ag/NaTaO_3_:Ba in the presence of a basic additive is shown in Figure 3B. It was confirmed that not HCO_3_^–^ or CO_3_^2–^ but a hydrated CO_2_ molecule is a reactant in photocatalytic CO_2_ reduction as in electrochemical CO_2_ reduction. HCO_3_^–^ functions as a buffer for supply of hydrated CO_2_ molecules. After the CO_2_ adsorbs on the Ag-cocatalyst to make CO_2_^•–^_(ad)_, CO evolves through path A (hydrogenation of CO_2_^•–^_(ad)_) or path B (reduction of COOH_(ad)_). Water is photooxidized to form O_2_ on the photocatalyst surface. Thus, adding a basic salt is key for efficient photocatalytic CO_2_ reduction with smooth supply of hydrated CO_2_ molecules.

### 3.3 Ag Cocatalyst-Loaded Photocatalysts for Single Particulate Photocatalytic CO_2_ Reduction to Form CO

Various metal oxide photocatalysts with different components and crystal structure have been developed for CO_2_ reduction based on loading Ag cocatalyst and adding NaHCO_3_ strategies from our group as shown in Table 2, for example, CaTa_4_O_11_,^22^ LaTa_7_O_19_,^22^ and KCaSrTa_5_O_15_^23,24^ photocatalysts. In addition, many metal oxide photocatalysts with wide band gaps have been reported for CO_2_ reduction such as La_2_Ti_2_O_7_,^25^ CaTiO_3_,^26^ SrTiO_3_:Al,^27^ Ga_2_O_3_:Zn,^28^ and ZnGa_2_O_4_/Ga_2_O_3_^29^ with the Ag cocatalyst from other groups. Substitution of elements is also a beneficial approach to develop new photocatalysts for CO_2_ reduction as well as for water splitting. For example, KCaSrTa_5_O_15_ (BG = 4.1 eV) has a tungsten bronze structure, which is similar to a defect type of perovskite structure (A_1–*x*_BO_3_). K, Ca, and Sr at an A site in KCaSrTa_5_O_15_ can be replaced with various other cations. Sr_*x*_K_*y*_Na_*z*_Ta_5_O_15_ and K_2_RETa_5_O_15_ (RE = rear earth metal) obtained by the substitution are also active for photocatalytic CO_2_ reduction.^30−32^

**Table 2:** Single Particulate Photocatalysts with Wide Band Gaps for CO_2_ Reduction Using Water as an Electron Donor^1,2,22−24^^a^

					activity [μmol h^–1^]			
photocatalyst	BG [eV]	crystal structure	Ag cocatalyst (wt %, loading method)	additive	H_2_	O_2_	CO	CO selectivity (%)
CaLa_4_Ti_4_O_15_	3.9	layered perovskite	Ag (1.0, LPR)	none	3.2	6.6	9.3	72
SrLa_4_Ti_4_O_15_	3.8	layered perovskite	Ag (1.0, LPR)	none	4.8	5.8	7.1	56
BaLa_4_Ti_4_O_15_	3.9	layered perovskite	Ag (1.0, LPR)	none	5.6	12	19	76
K_4_Nb_6_O_17_	3.4	layered	Ag(3.0, LPR)	NaHCO_3_	11	9	8	42
NaTaO_3_	4.0	perovskite	Ag(1.0, PD)	none	32	16	1.4	4.2
NaTaO_3_:Ba	4.1	perovskite	Ag(3.0, LPR)	NaHCO_3_	24	76	125	84
NaTaO_3_:Sr	4.1	perovskite	Ag(2.0, LPR)	NaHCO_3_	28	102	176	86
NaTaO_3_:Ca	4.1	perovskite	Ag(2.0, LPR)	NaHCO_3_	15	84	148	91
AgTaO_3_	3.4	perovskite	none	NaHCO_3_	27	15	4.2	13
KCaSrTa_5_O_15_	4.1	tungsten bronze	Ag(0.5, Imp)	NaHCO_3_	15	46	97	87
K_3_Ta_3_B_2_O_12_	4.0	tungsten bronze like	Ag(2.0, PD)	NaHCO_3_	55	32	16.7	23
SrTa_2_O_6_	4.4	CaTa_2_O_6_	Ag(3.0, LPR)	NaHCO_3_	95	86	87	48
BaTa_2_O_6_	4.1	CaTa_2_O_6_ as main phase	Ag(2.0, LPR)	NaHCO_3_	30	16	7	19
LaTa_7_O_19_	4.1	laminate	Ag(1.0, Imp)	NaHCO_3_	9	17	25	74
CaTa_4_O_11_	4.5	laminate	Ag(1.0, Imp)	NaHCO_3_	31	30	35	53

a Photocatalyst, 0.3–1.5 g; reactant solution, water (350–360 mL); flow gas, CO_2_ (1 atm); light source, 400 W high-pressure mercury lamp; reaction cell, inner irradiation quartz cell. PD, photodeposition; LPR, liquid-phase reduction; Imp, impregnation.

### 3.4 Rh–Ru Cocatalyst for CH_4_ Formation by Photocatalytic CO_2_ Reduction

Although many photocatalysts have been developed for CO_2_ reduction as mentioned above, obtained products are limited to two-electron reduction products such as CO and HCOOH. Therefore, it is challenging to demonstrate CO_2_ reduction to form CH_4_, an eight-electron reduction product, using water as an electron donor. Rh–Ru/NaTaO_3_:Sr(1%) continuously produces CH_4_, H_2_, and O_2_ under UV irradiation.^33^ The selectivity for CH_4_ formation based on the number of reacted electrons is about 10%. The e^–^/h^+^ ratio estimated from obtained products is 1.1, and TON based on CH_4_ formation with Rh and Ru cocatalysts is 2.0. No CH_4_ is obtained under Ar rather than CO_2_ flow. These results prove that CH_4_ is obtained by photocatalytic CO_2_ reduction using water as an electron donor over the Rh–Ru/NaTaO_3_:Sr(1%).

## 4 Z-Scheme CO_2_ Reduction Using Water as an Electron Donor under Visible Light Irradiation (Figure 1b)

It is a key issue to construct visible light responsive CO_2_ reduction system using water as an electron donor for efficient sunlight utilization beyond the wide band gap photocatalysts. In this section, visible light responsive photocatalysts for CO_2_ reduction in the presence of a sacrificial electron donor (Table 3) and application of those photocatalysts to Z-scheme systems for CO_2_ reduction using water as an electron donor under visible light (Table 4) are introduced.

**Table 3:** Sacrificial CO_2_ Reduction Using Metal Sulfide Photocatalysts under Visible Light Irradiation^19^^a^

				activity [μmol h^–1^]		
metal sulfide	crystal structure	BG, EG [eV]	electron donor	H_2_	CO	HCOOH
CuGaS_2_	chalcopyrite	2.3	K_2_SO_3_	11	0.25	trace
(AgInS_2_)_0.22_–(ZnS)_1.56_	wurtzite	2.3	Na_2_S + K_2_SO_3_	16	0.01	0
(AgInS_2_)_0.1_–(ZnS)_1.8_	wurtzite	2.6	Na_2_S	23	0.06	0.10
Ag_2_ZnGeS_4_	stannite	2.5	Na_2_S	38	0	0.14
ZnS:Ni(0.1%)	wurtzite + zinc blend	2.3	Na_2_S	22	trace	4.0
ZnS:Pb(1.0%)	wurtzite + zinc blend	2.4	Na_2_S	47	0.02	0.96
(ZnS)_0.9_–(CuCl)_0.1_	zinc blende	2.9	Na_2_S	140	0.01	0
ZnGa_0.5_In_1.5_S_4_	layered	2.7	Na_2_S	14	0.01	0

a Photocatalyst, 0.2–0.3 g; reactant solution, 0.05–0.1 mol L^–1^ Na_2_S or 0.1 mol L^–1^ K_2_SO_3(aq)_ (120–150 mL) or both; gas, CO_2_ (1 atm); light source, 300 W Xe lamp (λ > 420 nm); irradiation area, 33 cm^2^. BG = band gap, EG = energy gap.

**Table 4:** Z-Scheme Photocatalyst Systems for CO_2_ Reduction Using Water as an Electron Donor under UV or Visible Light Irradiation^3,4,19,43,46^^a^

					activity [μmol h^–1^]				
entry	reducing photocatalyst	O_2_-evolving photocatalyst	mediator	additive (mmol L^–1^)	H_2_	O_2_	CO	CO selectivity (%)	e^–^/h^+^
1	CuGaS_2_	RGO–TiO_2_	RGO	none	28.8	11.2	0.15	0.5	1.29
2	CuGaS_2_	RGO–(CoO_*x*_/BiVO_4_)	RGO	none	3.1	1.3	0.04	1.3	1.21
3	Cu_0.8_Ag_0.2_GaS_2_	RGO–(CoO_*x*_/BiVO_4_)	RGO	NaHCO_3_ (1)	4.0	1.6	0.03	0.7	1.26
4	CuGa_0.8_In_0.2_S_2_	RGO–(CoO_*x*_/BiVO_4_)	RGO	NaHCO_3_ (1)	3.5	1.6	0.04	1.1	1.11
5	(CuGa)_0.5_ZnS_2_	RGO–(CoO_*x*_/BiVO_4_)	RGO	NaHCO_3_ (1)	3.5	1.9	0.4	11	1.04
6	(CuGa)_0.5_ZnS_2_	RGO–(CoO_*x*_/BiVO_4_)	RGO	NaHCO_3_ (10)	12.0	6.4	1.8	13	1.08
7	(CuGa)_0.5_ZnS_2_	RGO–(CoO_*x*_/BiVO_4_)	RGO	KHCO_3_ (10)	8.1	4.6	2.1	20	1.11
8	(CuGa)_0.5_ZnS_2_	RGO–(CoO_*x*_/BiVO_4_)	RGO	NaHCO_3_ (100)	8.9	3.5	3.2	26	1.73
9	[Ru(dpbpy)]/(CuGa)_0.3_Zn_1.4_S_2_	BiVO_4_	Co[(tpy)_2_]^3+/2+^	NaHCO_3_ (250)	1.7	0.8	2.7	56	3.00
10	SrTiO_3_:Rh	BiVO_4_	none	none	8.7	4.0	0.018	0.2	1.09
11	Au/SrTiO_3_:Rh	BiVO_4_	none	none	3.5	1.9	0.031	0.9	0.93

a Photocatalyst, 0.1–0.4 g; reactant solution, water (120–150 mL); flow gas, CO_2_ (1 atm); light source, 300 W Xe lamp (λ > 300 nm for TiO_2_ and λ > 420 nm for BiVO_4_ systems); irradiation area, 33 cm^2^.

### 4.1 Visible-Light Responsive Metal Sulfide Photocatalysts for CO_2_ Reduction Using Sacrificial Electron Donor

Metal sulfide photocatalysts are active for not only water reduction but also CO_2_ reduction under visible light using a sacrificial electron donor. For example, CdS is active for sacrificial CO_2_ reduction to form CO in an aqueous solution containing a sacrificial reagent.^34,35^ Metal sulfides with various crystal structures have also been developed for sacrificial CO_2_ reduction under visible light irradiation as shown in Table 3.^19^ CuGaS_2_ and ZnS:Ni photocatalysts are highly active for CO and HCOOH formation, respectively. However, these CO_2_ reductions are not artificial photosynthesis because strong sacrificial electron donors are used. Since they cannot oxidize water into O_2_ because of self-photooxidation (photocorrosion), single particulate overall water splitting and CO_2_ reduction accompanied by O_2_ evolution by water oxidation as shown in Figure 1a is difficult. Construction of Z-scheme systems is a beneficial approach to employ metal sulfide photocatalysts showing CO_2_ reduction activity combined with an O_2_-evolving photocatalyst as shown in Figure 1b.

### 4.2 Z-Scheme System Employing RGO as a Solid-State Electron Mediator (Figure 4A(a))

A Z-scheme system consisting of CuGaS_2_ as a reducing photocatalyst, TiO_2_ as an O_2_-evolving photocatalyst, and reduced graphene oxide (RGO) as a solid-state electron mediator is active for not only water splitting^36^ but also CO_2_ reduction to form CO (Table 4, entry 1).^19^ The carbon source for the CO_2_ reduction product should carefully be checked, because RGO is a carbon material. ^13^CO formed under ^13^CO_2_ flow, indicating that flowed CO_2_ was the carbon source. However, ^12^CO was obtained in addition to the ^13^CO. Moreover, a small amount of CO formed even under Ar gas instead of CO_2_. So, a part of CO formed by Z-scheme CO_2_ reduction, whereas other CO formed by photooxidation of RGO on TiO_2_. The Z-scheme system works only under UV light because of limitations of TiO_2_. When visible light responsive RGO–(CoO_*x*_/BiVO_4_) is employed instead of RGO–TiO_2_, Z-scheme CO_2_ reduction to form CO proceeds using water as an electron donor under visible light in an aqueous suspension (Table 4 entry 2).^3^ CO is not obtained under Ar flow in the Z-scheme system composed of RGO–(CoO_*x*_/BiVO_4_) unlike that using RGO–TiO_2_. The inhibition of RGO oxidation is due to less oxidation power of holes photogenerated in the valence band of BiVO_4_ than that of TiO_2_.

**Figure 4:**
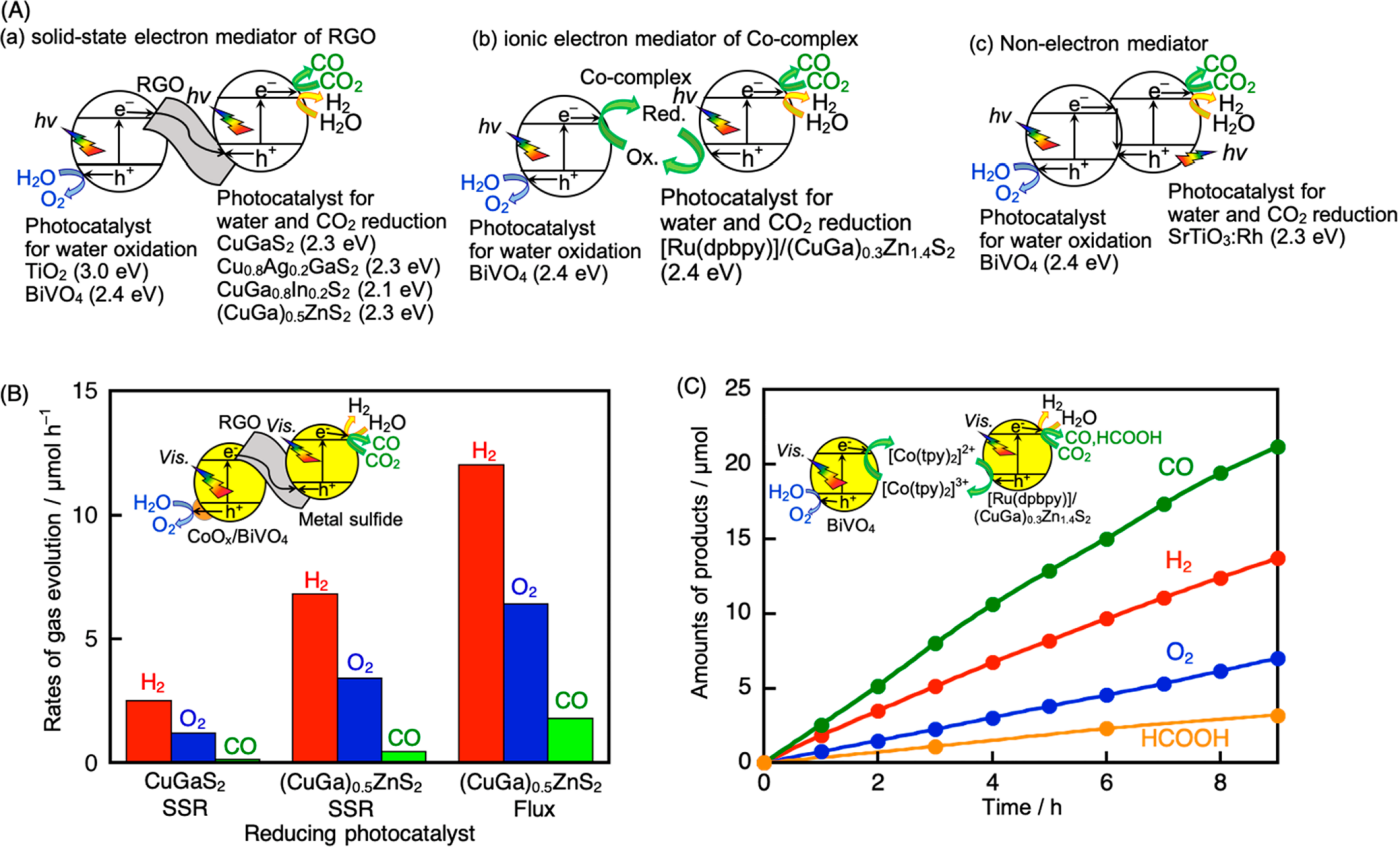
(A) Various types of Z-scheme photocatalysts for CO_2_ reduction using water as an electron donor. (B) Z-scheme CO_2_ reduction under visible light irradiation using CuGaS_2_ or (CuGa)_0.5_ZnS_2_ prepared by a SSR or a flux method combined with RGO–(CoO_*x*_/BiVO_4_). Reproduced with permission from ref (4). Copyright 2022 American Chemical Society. (C) Z-scheme CO_2_ reduction under visible light irradiation using [Ru(dpbpy)]/(CuGa)_0.3_Zn_1.4_S_2_, BiVO_4_, and [Co(tpy)_2_]^3+/2+^. Reproduced with permission from ref (43). Copyright 2018 The Royal Society of Chemistry. Photocatalyst, 0.1–0.4 g; reactant solution, NaHCO_3(aq)_ (120–150 mL); flow gas, CO_2_ (1 atm); light source, 300 W Xe lamp (λ > 420 nm); irradiation area, 33 cm^2^.

Making a solid solution based on CuGaS_2_ with p-type character is beneficial to developing a reducing photocatalyst, because the band structure is tunable by a change in the composition of the solid solution.^6,37^ For example, solid solutions of CuGaS_2_ with CuInS_2_ can absorb longer wavelengths of visible light than CuGaS_2_, because In 5s5p orbitals of CuInS_2_ lower the conduction band consisting of Ga 4s4p orbitals of CuGaS_2_ resulting in band gap narrowing. Red-powdered CuGa_0.8_In_0.2_S_2_, which absorbs visible light up to 600 nm functions as a CO_2_-reducing photocatalyst in the Z-scheme system (Table 4, entry 4). Making a (CuGa)_1–*x*_Zn_2*x*_S_2_ solid solution between CuGaS_2_ and ZnS improves CuGaS_2_ performance, though the band gap does not become narrower than that of CuGaS_2_.^38^ The Z-scheme system using (GuGa)_0.5_ZnS_2_ prepared by a solid-state reaction (SSR) combined with RGO–(CoO_*x*_/BiVO_4_) shows higher water splitting and CO_2_ reduction activities than that using CuGaS_2_ prepared by SSR (Figure 4B). When the (CuGa)_0.5_ZnS_2_ particle is prepared by a flux method, fine particles of (CuGa)_0.5_ZnS_2_ with a few hundreds of nanometers in size are obtained, while the particle size when prepared by conventional SSR is about 1 μm.^39^ When the fine particulate (CuGa)_0.5_ZnS_2_ is applied to a Z-scheme system, photocatalytic water splitting and CO_2_ reduction are much enhanced (Figure 4B).^4^ The Z-scheme CO_2_ reduction activity strongly depends on the reactant solution conditions (Table 4, entries 5–8). Addition of a basic salt not only stabilizes but also enhances Z-scheme CO_2_ reduction because of efficient supply of hydrated CO_2_ to the photocatalyst surface. We stress that the selectivity for CO formation in the Z-scheme CO_2_ reduction reaches 10–20% even using bare metal sulfide without surface modification. Although a Ag cocatalyst is effective for CO_2_ reduction to form CO over wide band gap metal oxides as mentioned in section 3, Ag on a metal sulfide does not enhance CO formation in the Z-scheme CO_2_ reduction at the present stage, probably due to poisoning of the Ag surface by sulfurization. Therefore, further highly selective CO_2_ reduction is expected by introducing a suitable active site and surface modification of the metal sulfide photocatalyst for Z-scheme CO_2_ reduction.

### 4.3 Z-Scheme System Employing a Co-Complex as an Electron Mediator (Figure 4A(b))

Metal complexes have been widely examined as selective CO_2_-reducing catalysts in electrochemistry, coordination chemistry, and photochemistry.^40,41^ Recently, hybrid systems combining a metal complex catalyst with semiconductor photocatalyst materials have been studied for highly selective CO_2_ reduction in photoelectrochemical and photocatalytic systems.^42^ For example, Z-scheme CO_2_ reduction under visible light has been demonstrated using [Ru(dpbpy)]-loaded (CuGa)_0.3_Zn_1.4_S_2_, BiVO_4_, and a Co-complex as an electron mediator (Figure 4C).^43^ CO evolves as a main reduction product with introduction of the highly active Ru-complex catalyst for CO_2_ reduction on (CuGa)_0.3_Zn_1.4_S_2_. HCOOH is also produced in the reaction. The catalytic activity of a metal complex is usually inhibited in the presence of O_2_. Therefore, it is notable that CO_2_ reduction and simultaneous O_2_ evolution proceed even using a metal complex catalyst with a semiconductor photocatalyst in an aqueous solution, though the amount of O_2_ is small compared with a stoichiometric amount.

### 4.4 Z-Scheme System Driven by Interparticle Electron Transfer without an Electron Mediator (Figure 4A(c))

SrTiO_3_:Rh shows high sacrificial H_2_ evolution activity, though it does not oxidize water into O_2_.^44^ However, SrTiO_3_:Rh can be employed to construct a Z-scheme system working via interparticle electron transfer with BiVO_4_ without an electron mediator (Figure 4A(c)).^45,46^ The Z-scheme system reduces CO_2_ to CO accompanied by H_2_ and O_2_ under visible light. Loading Ag or Au cocatalyst on SrTiO_3_:Rh improves the CO evolution activity (Table 4, entries 10, 11). The suitable pH is around 4, because SrTiO_3_:Rh and BiVO_4_ particles aggregate well with each other to get good contact between the particles, resulting in smooth electron transfer from BiVO_4_ to SrTiO_3_:Rh via interparticle electron transfer. It is notable that the Z-scheme CO_2_ reduction proceeds using just photocatalyst powders, water, and CO_2_ because of self-pH-adjustment by dissolved CO_2_.

## 5 CO_2_ Reduction on p-Type Cu(I)-Containing Metal Sulfide Photocathodes under Visible Light Irradiation (Figure 1c,d)

**Figure 5:**
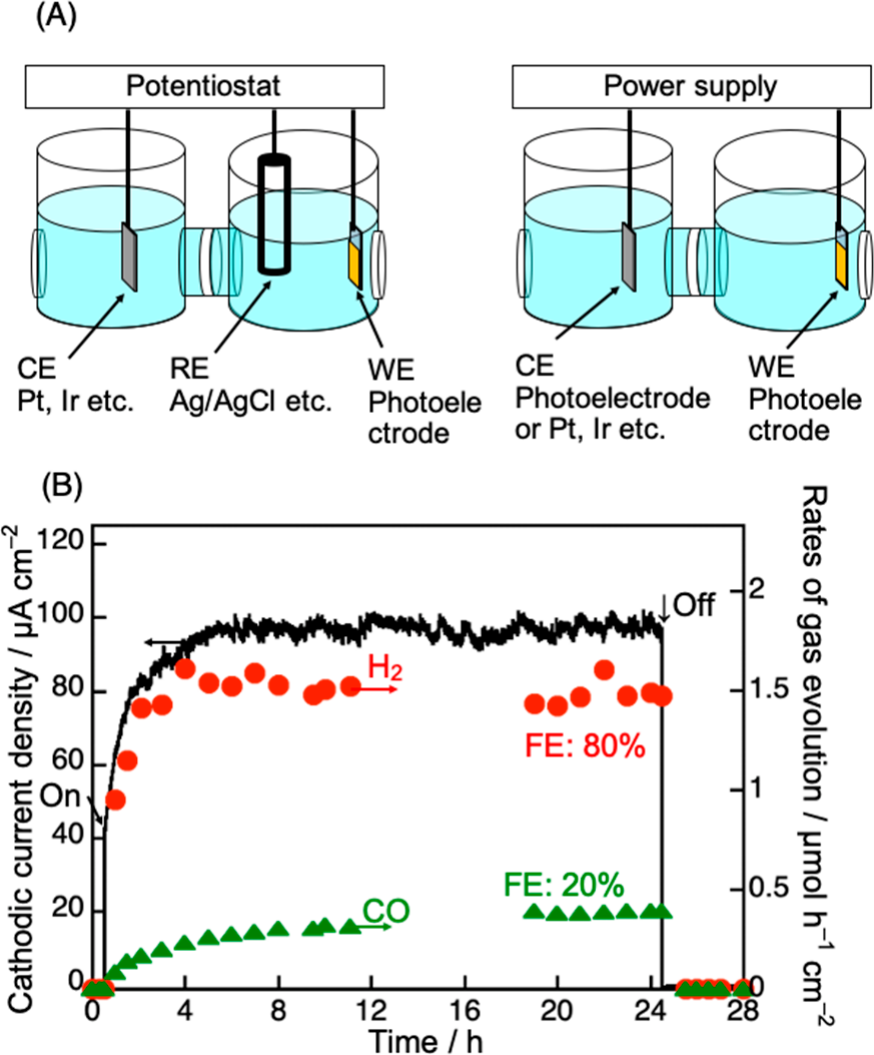
(A) Two-electrode and three-electrode systems for photoelectrochemical CO_2_ reduction. (B) Photoelectrochemical CO_2_ reduction under visible light irradiation over a (CuGa)_0.5_ZnS_2_ powder-based photocathode. Electrolyte, 0.1 mol L^–1^ KHCO_3(aq)_; flow gas, CO_2_ (1 atm); light source, 300 W Xe lamp (λ > 420 nm); applied bias, 0.1 V vs RHE (−0.5 V vs Ag/AgCl (pH 6.9)). Reproduced with permission from ref (4). Copyright 2022 American Chemical Society.

A photoelectrochemical CO_2_ reduction system is also interesting to construct an artificial photosynthesis system. Photoelectrochemical measurement is generally conducted in a 3-electrode system or a 2-electrode system connected to a potentiostat and a power supply (Figure 5A). Scientifically intrinsic information on the working electrode, for example, an absolute electrode potential, is obtained with the 3-electrode system using a reference electrode. The 2-electrode system is useful for evaluation of cell performance such as open circuit voltage, short circuit current, and energy conversion efficiency. It is meaningless in a photoelectrochemical cell if an externally applied voltage is larger than the theoretical voltage of electrolysis, for example, 1.23 V for water splitting. Applying no external bias is ideal. To compare the performance of a photoelectrode, a current–potential curve is usually measured using the 3-electrode system. In addition, analysis of products by bulk electrolysis is also indispensable, as well as measurement of photocurrent to examine the Faradaic efficiency, that is, electrochemical selectivity. The Faradaic efficiency reveals if the photocurrent is due to desired redox reactions. Moreover, not only the Faradaic efficiency but also a partial photocurrent density (i.e., rate of production) are important to see how fast a certain product is formed. Incident photon to current conversion efficiency (IPCE) and solar energy conversion efficiency are also important.

The photoelectrochemical cell can employ p-type semiconductors as a photocathode even photocorrosive materials. For example, visible light responsive CuGaS_2_,^47^ (CuGa)_0.5_ZnS_2_,^4,38^ Cu_0.8_Ag_0.2_GaS_2_,^48,49^ and Cu_2_ZnGeS_4_^50^ function as a CO_2_-reducing photocathodes. The bare (CuGa)_0.5_ZnS_2_ photocathode reduces CO_2_ to CO with high stability under visible light with application of an external bias (Figure 5B).^4^ Faradaic efficiencies for CO and H_2_ formation are 20% and 80%, respectively, being almost 100% of total Faradaic efficiency. It is stressed that high CO formation is observed even without cocatalyst and surface modification on the photocathode.

Surface modification with CdS and ZnS of an n-type semiconductor and loading of a cocatalyst improve the performance of p-type Cu_0.8_Ag_0.2_GaS_2_,^49^ Cu_2_ZnGeS_4_,^51^ and (CuGa_1–*y*_In_*y*_)_1–*x*_Zn_2*x*_S_2_ solid solution^52^ photocathodes. Introduction of an electrically conducting polymer such as polypyrrole (PPy) or poly(3,4-ethylenedioxythiophene) (PEDOT) as hole transporter also improves a photocathode composed of a powdered material, because electric contact between the powders and the substrate electrode such as FTO is usually poor.^53,54^ PPy-modified CuGaS_2_ gives higher cathodic photocurrent for water and CO_2_ reduction than a bare CuGaS_2_ photocathode. Moreover, the 2-electrode system combining a PEDOT–CuGaS_2_ photocathode and a CoO_*x*_/BiVO_4_ photoanode with visible light response also reduces CO_2_ to CO using water as an electron donor under application of a small bias and simulated sunlight irradiation.

## 6 Conclusions and Perspectives

Artificial photosynthesis is ideal green chemistry and technology to convert and store solar energy to chemical products as an uphill reaction. Solar water splitting to produce H_2_ is representative of artificial photosynthesis. Solar water splitting using a powder-based photocatalyst on a large scale (100 m^2^) has been demonstrated.^5^ It will accelerate the industrial application of solar hydrogen production in the near future. In contrast to solar water splitting, artificial photosynthetic CO_2_ utilization using photocatalysts is still at the stage of basic research. However, recent and rapid progress of this research area is hopeful. A variety of photocatalyst and photoelectrode systems for CO_2_ utilization has been extensively developed using homogeneous and heterogeneous photocatalyst materials. This Account focused on photocatalytic and photoelectrochemical systems based on particulate photocatalysts for CO_2_ reduction as an artificial photosynthesis system working under UV and visible light.

Highly active photocatalysts for water splitting such as BaLa_4_Ti_4_O_15_ (BG = 3.9 eV) and doped NaTaO_3_ (BG = 4.1 eV) were able to be applied to CO_2_ reduction, because they have sufficiently high conduction bands and enough potential for water oxidation to form O_2_. The O_2_ evolution ability and a suitable cocatalyst working as a reaction center for CO_2_ reduction are indispensable for photocatalytic CO_2_ reduction using water as an electron donor. Ag and Rh–Ru cocatalysts were developed for CO and CH_4_ formation, respectively. Moreover, the photocatalytic activity was increased with optimization of reaction conditions such as tuning of the reactant solution. Metal sulfide photocatalysts with a high conduction band and visible light response are attractive for CO_2_ reduction, though they cannot oxidize water. This means that the metal sulfide photocatalyst itself cannot use water as an electron donor to achieve an uphill reaction. However, CuGaS_2_, (CuGa)_1–*x*_Zn_2*x*_S_2_, and CuGa_1–*x*_In_*x*_S_2_ metal sulfide materials were able to be employed as a CO_2_-reducing photocatalysts to make a Z-scheme photocatalyst system to achieve photocatalytic CO_2_ reduction using water as an electron donor under visible light irradiation. p-Type metal sulfides CuGaS_2_, (CuGa)_1–*x*_Zn_2*x*_S_2_, and Cu_1–*x*_Ag_*x*_GaS_2_ were able to be applied to a photocathode for photoelectrochemical CO_2_ reduction, even if their powdered materials were employed.

Strategies to design photocatalytic and photoelectrochemical systems for CO_2_ reduction using water as an electron donor under visible light irradiation become clearer as mentioned above. Therefore, it is expected that more efficient photocatalyst and photoelectrode systems can be developed with further extensive study. We believe that photocatalyst and photoelectrode systems for solar CO_2_ utilization can be a practical use in the future as well as solar hydrogen production by water splitting.
